# European Veterinary Institutions1Read before the annual meeting of the American Veterinary Medical Association, New York, September, 1899.

**Published:** 1899-11

**Authors:** James B. Paige

**Affiliations:** Amherst, Mass.


### EUROPEAN VETERINARY INSTITUTIONS.[1]

By James B. Paige, D.V.S.,
AMHERST, MASS.

(Concluded from page 625.)

As previously mentioned, the buildings and equipment at Bern and Budapest are entirely modern. At the former institution there are four large buildings, consisting of an administration building, forge and workshop, stable, and the anatomical and pathological building, containing lecture-room, dissecting-room, laboratories, etc. These four buildings are arranged on four sides of a square facing a central court, which contains a covered enclosure, paddocks, and an exercising ring.

The location, buildings, and equipment of the Hungarian Veterinary College at Budapest are unequalled by any similar institution in all Europe. Its grounds are located in the very heart of the most cosmopolitan city on the Continent. Its buildings are all new, built of the finest material, and are in every respect complete for the purposes for which they were erected. The hospital is a model for a building of such a character. The main part is divided into separate sections for the complete separation of animals suffering from different diseases; there is an independent surgical ward in the rear; a glander section upon the left; the pneumonia patients are consigned to a compartment upon the right, while animals suffering with colic and other non-contagious dis-

[1] Read before the annual meeting of the American Veterinary Medical Association, New York, September, 1899.

eases are provided for in another portion of the building. In those sections used for animals suffering with contagious diseases everything has been arranged so as to lessen the liability of spreading infection and to allow of easy and complete disinfection; mangers of stone are used; the walls above are of glazed tiles, the floors of cement, with provisions for perfect drainage; the partitions and walls are built of brick, with numerous windows and openings for the admission of light and air.

The bacteriological laboratory is a large two-story building of brick, of handsome design, finished and furnished in a most elaborate manner. An L connected with it serves as an isolation ward for the confinement of dogs suspected of rabies.

The pathological, chemical, and physiological laboratories are equal in every respect to the one just described. An ideal college is that at Budapest, but no place for an American to study, as all instruction is given in the Hungarian language.

The course of study is quite uniform in all Continental institutions. This applies especially to all German colleges, which are under the direction of the boards of education. Students in attendance at one school for a time in going to another are given full credit for all examinations passed in that first attended. The curriculum of the Royal Veterinary Institution of Stüttgart gives an idea of the required course of study at nearly all of the colleges. There may be slight variation in this respect, or it may be that some particular subject is given more attention at one college than at another. The course at Stüttgart includes: General consideration of veterinary science; physics; chemistry—organic and inorganic—with practical work, botany; zoölogy; anatomy of domestic animals, with practical exercises; histology, with practical exercises; general and special physiology; embryology; zoötechnics; hygiene and dietetics; lectures upon the exterior of the horse and other draught animals; theory of horseshoeing, with practical work; general pathology; general and special pathological anatomy; autopsies and pathological demonstrations; histopathological exercises; parasitology, with exercises; pharmacology; pharmaceutical chemistry; practical exercises in pharmacy; materia medica, with prescription writing; therapeutics; special pathology and therapeutics; general and special surgery; ophthalmology, with practical work with the ophthalmoscope; operative surgery, with practical work; obstetrics; medical and surgical clinics; clinic for cattle, sheep, and swine; jurisprudence; exercises in the preparation, opinions, and reports; veterinary police; infectious diseases and bacteriology ; theoretical and practical meat-inspection ; history of veterinary science ; the science of agriculture as applied to soil-cultivation, plant-production, etc. To this list might be added the latest course introduced into the curriculum of the Munich school, viz., pisciculture and pathology.

With a few exceptions, either three and one-half or four years of study are required to complete the work. The year is divided into two semesters. The student year begins with the winter semester, which opens about October 15th, continuing until March 15th. Then comes a vacation, lasting usually until the first Tuesday after Easter Sunday. The summer semester continues until August 1st. In some colleges senior students are required to do hospital work during one-half of the vacation time. The Brussels school and that at Lemberg, in the extreme northeastern portion of Austria, near the Russian border, require only three years' attendance. In the latter institution the instruction is given in the Polish language.

The number of students in attendance at the different institutions varies greatly, depending upon the size and population of the State in which the college is located or the advantages offered in special lines of work. They are divided into two classes—civil and military—to which might be added a third, called "hospitanten," who are graduates from other veterinary institutions or agricultural colleges, foreigners, etc., who attend to take special courses.

During 1895 and 1896, Budapest had in attendance 300 students, 50 of whom were military. Berlin had 500, 125 of whom were military. Lyons had 200. In 1894 and 1895, Munich had in all classes during the winter semester 198 students, and in the summer semester 181. During the year 28 students took the final examination for graduation, only 18 of whom received certificates. Not in the least discouraged, the unfortunate one usually resumes work with the regular classes, and after a certain time are again permitted to present themselves for final examinations.

One may get a general idea of the expense of attending a school in Germany if we take the tuition-fee charged at Stüttgart as an example. The price necessarily varies in different places, but these given are a fair average. For the regular German students 30 marks ($7.50) per semester is the tuition fee for the purely theoretical instruction. The price for foreigners for the same courses is 50 marks ($12.50). For laboratory work of all kinds an extra fee is paid, according to the number of exercises per week. Where there are from one to four hours per week the fee is 10 marks ($2.50) per semester; five to six hours, 15 marks ($3.75); for more than six hours, 20 marks ($5.00). Transient students (hospitanten) pay for partial lecture courses the same prices as charged regular students for laboratory exercises, depending upon the number of lectures per week. The tuition to all clinics, 20 marks ($5.00) per semester. In the Munich college in 1895 and 1896 the total tuition charged veterinarians, including all lectures and practical work in bacteriology and pathology and admission to both medical and surgical clinics, was $8.50 per semester.

The curricula of all Continental schools provide for instruction in both theoretical and practical work. Tests are given at the final examinations. If a prospective graduate in his examination makes a diagnosis of sarcoptic mange, he must demonstrate the presence of the parasite with the microscope. If his diagnosis is azoturia, he must prove by chemical test, to the satisfaction of the examiner, that hæmatin is present in the urine, etc. This method of combining the theoretical and practical is carried into all hospital work. When assigned a patient the student is expected to make and confirm his diagnosis and suggest a course of treatment. He is also closely questioned on the pathology of the disease and its probable termination. Each day he must stand an examination on the condition of the animal at that time. If an ordinary surgical case, the students perform the operation in the presence of the professor in charge of the clinic. Before being allowed to graduate the student must have had charge of a given number of medical cases and have performed certain surgical operations.

Figures being more convincing than words, let me give a few with reference to the amount of clinical material furnished the students at one or two of the colleges. In 1888 at Zurich, the capital of the Swiss canton of that name, which has, including its eleven suburbs, a population of 126,499, there were brought into the internal clinic of the veterinary college for treatment 886 horses, 22 cattle, 4 goats, 449 dogs, 20 cats, 3 birds, and 1 swine, making a total of 1379 domestic animals treated in that department for the year. External clinic (ambulatorische): Horses, 2371; cattle, 905; dogs, 56; swine, 48; sheep and goats, 4; cats, 2; total, 3386. Consultations in hospital: Horses, 1960; cattle, 43; swine, 18; sheep, 11; dogs, 726; cats, 83; birds, 6; goat, 1; total, 2848. Autopsies: Horses, 53: cattle, 5; swine, 3; goats, 4; dogs, 52; cats, 16; rabbits, 3; birds, 85; fish, 2; slaughter-house material, 40; contributed specimens, 81. Total, 343 objects. Making a grand total of 7956 cases presented for examination in one year. In the last number of the *Monatshefte für Praktische Theirheilkunde* (vol. x., No. 12) are given the statistics of the surgery clinic of the Berlin hospital for the years 1898–99: During this time 753 horses were admitted to this clinic; 369 operations performed in which anæsthetics were administered 87 times. There were sent to the pathological department of the Munich school by veterinarians in Bavaria during the year 1894–95 no less than 265 specimens for examination and demonstration purposes. This does not include the material obtained from the different departments of the institution. Into the different clinics there were admitted during the same time 821 horses, 13 ruminants, 2361 dogs, 117 cats, and 16 birds. During the year 1895, 10,000 patients were received and given either medical or surgical treatment at the small-animal clinic at Berlin. This number included dogs, cats, rabbits, parrots, canaries and other song-birds, and pigeons.

One finds a great variety of material for study at the various clinics. Diseases which are never seen on this continent are to be observed there almost daily; especially is this true of many of the parasitic diseases. This is doubtless due to the fact that the animal population is older and much more concentrated than with us—conditions which are favorable for the spread and propagation of parasitic and bacterial disorders.

Of the Continental teachers and veterinarians I feel that I need say but little, as I am sure most of us have already gained an acquaintance with them through their works. They have had in the past great advantages, and have made the most of them. We find among their numbers specialists in every department of veterinary science, who are recognized authorities in their respective lines of work. They and their predecessors have built up some of the best veterinary institutions in the world, and have added very largely to our collection of veterinary literature, and as a result of their labors they are able to suppress, eradicate, and prevent those animal diseases which have in times past swept over all Europe, destroyed their livestock, bringing poverty to the people, and endangered their lives.

To one contemplating a trip abroad for study let me offer this advice: Before you go make sure that you know what you want, and ascertain where you can get the best in that particular line of work. All institutions do not offer the same advantages for special study. At some of the smaller colleges one may get better instruction in a certain special line of work than can be obtained at one of the larger ones.

Europeans are, as a rule, very conservative, adhering with great tenacity to old methods and customs, giving up the old for the new only after having proven their superiority by long-continued trial and application.

Above all, do not go abroad for information on subjects pertaining to veterinary science until you have exhausted the resources of one of your own institutions.

